# Clinical and genetic features of Koreans with retinitis pigmentosa associated with mutations in rhodopsin

**DOI:** 10.3389/fgene.2023.1240067

**Published:** 2023-08-29

**Authors:** Young Hoon Jung, Jay Jiyong Kwak, Kwangsic Joo, Hyuk Jun Lee, Kyu Hyung Park, Min Seok Kim, Eun Kyoung Lee, Suk Ho Byeon, Christopher Seungkyu Lee, Jinu Han, Junwon Lee, Chang Ki Yoon, Se Joon Woo

**Affiliations:** ^1^ Department of Ophthalmology, Seoul National University College of Medicine, Seoul National University Bundang Hospital, Seongnam, Republic of Korea; ^2^ Institute of Vision Research, Department of Ophthalmology, Severance Eye Hospital, Yonsei University College of Medicine, Seoul, Republic of Korea; ^3^ Department of Ophthalmology, Seoul National University Hospital, Seoul, Republic of Korea; ^4^ Institute of Vision Research, Department of Ophthalmology, Gangnam Severance Hospital, Yonsei University College of Medicine, Seoul, Republic of Korea

**Keywords:** retinitis pigmentosa, rhodopsin, generalized retinitis pigmentosa, sector retinitis pigmentosa, Koreans

## Abstract

**Purpose:** To investigate the clinical features, natural course, and genetic characteristics of Koreans with rhodopsin-associated retinitis pigmentosa (*RHO*-associated RP).

**Design:** We conducted a retrospective, multicenter, observational cohort study.

**Participants:** We reviewed the medical records of 42 patients with *RHO*-associated RP of 36 families who visited 4 hospitals in Korea.

**Methods:** Patients with molecular confirmation of pathogenic variants of the *RHO* gene were included. The patients were divided into two subgroups: the generalized and sector RP groups. A central visual field of the better-seeing eye of <10° or a best-corrected visual acuity of the better-seeing eye <20/40 indicated the progression to late-stage RP.

**Results:** The mean age at which symptoms first appeared was 26.3 ± 17.9 years (range: 8–78 years), and the mean follow-up period was 80.9 ± 68.7 months (range: 6–268 months). At the last follow-up visit, the generalized RP group showed a significantly higher rate of visual field impairment progression to late-stage RP than that of the sector RP group (22 of 35 [62.9%] vs. 0 of 7 [0.0%], *p* = 0.003). No cases in the sector RP group progressed to generalized RP. Best-corrected visual acuity deterioration to late-stage RP was observed only in the generalized RP group (13 of 35 patients; 37.1%), whereas no deterioration was observed in the sector RP group. We identified 16 known and three novel *RHO* mutations, including two missense mutations (p.T108P and p.G121R) and one deletion mutation (p.P347_A348del). The pathogenic variants were most frequently detected in exon 1 (14 of 36 [38.9%]). The most common pathogenic variants were p.P347L and T17M (5 of 36 [13.9%] families). Among 42 patients of 36 families, 35 patients of 29 families (80.6%) presented with the generalized RP phenotype, and seven patients of seven families (19.4%) presented with the sector RP phenotype. Three variants (p.T17M, p.G101E, and p.E181K) presented with both the generalized and sector RP phenotypes.

**Conclusion:** This multicenter cohort study provided information on the clinical and genetic features of *RHO*-associated RP in Koreans. It is clinically important to expand the genetic spectrum and understand genotype-phenotype correlations to ultimately facilitate the development of gene therapy.

## 1 Introduction

Retinitis pigmentosa (RP), the most common type of progressive inherited retinal dystrophy (IRD), is characterized by rod and cone photoreceptor degeneration (Hartong et al., 2006; Verbakel et al., 2018). RP occurs in approximately 1 of each 3,500 individuals with the following inheritance patterns: autosomal dominant (15%–20%), autosomal recessive (20%–25%), and X-linked (10%–15%) (Wang et al., 2005). The genetic and clinical features of RP are somehow similar to those of other IRDs, such as cone-rod dystrophy, macular dystrophies, Leber congenital amaurosis, and congenital stationary night blindness (Zhang, 2016). Therefore, an accurate diagnosis via gene sequencing is crucial.

Since a rhodopsin (*RHO*) gene mutation, p.P23H, was initially reported, >230 different mutations have been reported to be associated with RP, accounting for 25%–30% of all autosomal dominant RP cases (Liu et al., 2021). The common phenotypes in *RHO* mutations are generalized (classical) and sector RP. Clinical features and visual prognosis differ between patients with generalized and sector RP. Although sector RP progresses relatively slower than generalized RP, several reports stated that it eventually progresses to the generalized type (Ballios et al., 2021; Nguyen et al., 2021). However, the difference in genetic and clinical features between generalized and sector RP is unclear. Although there have been studies on *RHO-*associated RP in Caucasian, Japanese, and Chinese populations, no such studies have been conducted on Koreans.

Thus, our study aims to identify the clinical features, including natural course, pathogenic variants, and genotype-phenotype correlations, in Koreans with *RHO*-associated RP.

## 2 Materials and methods

### 2.1 Patients and inclusion criteria

This retrospective observational cohort study was conducted at four tertiary centers: Seoul National University Bundang Hospital, Seoul National University Hospital, Severance Eye Hospital, and Gangnam Severance Hospital. An institutional Review Board approval was obtained (IRB no: B-2206-762-101), and the study was conducted in accordance with the principles of the Declaration of Helsinki. Initially, 876 patients diagnosed with RP via gene sequencing were screened. We enrolled patients with the clinical features of RP and one or more pathogenic variants of *RHO*. Finally, 42 patients (42 of 876; 4.8%) from 36 families were included.

### 2.2 Genetic analyses

A comprehensive custom gene panel of 295 known and candidate genes or a 429 gene-targeted panel linked to IRDs was used for genetic analyses, as reported in previous studies (Seong et al., 2015; Kim et al., 2019; Kim et al., 2021). Targeted next-generation sequencing (Targeted NGS, Illumina NextSeq 550 system; San Diego, CA, USA) or whole exome sequencing (WES; Illumina NovaSeq 6000 system) were performed. Targeted NGS was performed using custom-designed RNA oligonucleotide probes and a target enrichment kit (Celemics, Seoul, South Korea). WES was performed using the xGen Exome Research Panel v1.0 (Integrated DNA Technologies, Inc., Coraville, IA, USA) and SureSelect Human All Exon v6 enrichment kit (Agilent Technologies, Santa Clara, CA, USA).

### 2.3 Clinical data collection and classification

We retrospectively reviewed the medical records, and the patients were divided into two subgroups: the generalized and sector RP groups. Ophthalmic examinations included best-corrected visual acuity (BCVA), refraction, intraocular pressure (IOP), wide-field color fundus photography (Optos PLC, Dunfermline, UK), spectral domain OCT (Heideiberg Engineering, Heidelberg, Germany; Carl Zeiss Meditec, Germany), full-field electroretinography (ERG), and Goldmann visual field. ERG was performed based on the standards of the International Society for Clinical Electrophysiology of Vision, available at www.iscev.org. Based on the criteria of the World Health Organization, the International Classification of Diseases 11 classifies vision impairment into five groups: no visual impairment (BCVA ≥20/40), mild visual impairment (20/70 ≤ BCVA <20/40), moderate visual impairment (20/200 ≤ BCVA <20/70), severe visual impairment (20/400 ≤ BCVA <20/200), and blindness (BCVA <20/400). As many patients with RP maintain relatively good visual acuity until the late stage, we considered a decline in BCVA to <20/40 in the better-seeing eye to be an indication of progression to late-stage RP. Moreover, late-stage RP was identified when the central VF of the better-seeing eye was <10°, as VF loss is relatively an early symptom of RP.

*RHO* mutations are classified in HGMD (http://www.hgmd.cf.ac.uk/) based on the experimentally studied cellular and biochemical characteristics: class 1 (post Golgi trafficking and outer segments targeting), class 2 (misfolding and endoplasmic reticulum retention and instability), class 3 (disrupted vesicular traffic and endocytosis), class 4 (altered post-translational modifications and reduced stability), class 5 (altered transduction activation), class 6 (constitutive activation), class 7 (dimerization deficiency), and unclassified (Athanasiou et al., 2018).

We also ranked and scaled the combined annotation dependent depletion (CADD) score, an *in silico* prediction tool. A scaled CADD score greater than 20 indicating a variant in the top 1% of deleterious variants in the human genome, while a score greater than 30 indicating a variant in the top 0.1%.

### 2.4 Statistical analysis

Statistical analyses were performed using the SPSS version 27.0 software (SPSS Inc., Chicago, Illinois, USA). Either the χ^2^ test or Fisher’s exact test was used to compare the categorical variables between the two groups, and the Mann-Whitney U test was used to compare the numerical variables. The Pearson correlation test was performed to evaluate the correlation between CADD score and the progression to late-stage RP.

## 3 Results

The demographic and clinical characteristics of the 42 patients of 36 families are listed in Table 1. Among 42 patients of 36 families, 35 patients of 29 families (80.6%) presented with the generalized RP phenotype, and seven patients of seven families (19.4%) presented with the sector RP phenotype. The mean age at which symptoms first appeared was 24.0 ± 17.9 years (range: 8–78 years) for generalized RP and 35.0 ± 16.9 years (range: 24–78 years) for sector RP (*p* = 0.100). The mean follow-up period was 85.9 ± 73.3 months (range: 6–268 months) for generalized RP and 58.9 ± 40.0 months (range: 6–108 months) for sector RP (*p* = 0.536). No significant differences between the two phenotypes were detected in terms of sex, age, follow-up period, refraction, IOP, or BCVA.

**TABLE 1:** Characteristics of patients with RHO-Associated RP.

Characteristics	Total (n = 42)	Generalized RP (n = 35)	Sector RP (n = 7)	*p*-value
Male: Female	20 : 22	17 : 18	3 : 4	1.000
Age (diagnosis)	39.0 ± 17.0	38.6. ± 16.8	46.7 ± 17.7	0.451
Age (symptom onset)	26.3 ± 17.9	24.0 ± 17.9	35.0 ± 16.9	0.100
Lens (phakic: pseudophakic)	69 : 15	57 : 13	12 : 2	1.000
Mean follow up period (month)	80.9 ± 68.7	85.9 ± 73.3	58.9 ± 40.0	0.536
Initial symptom (multiple choice)				
Night blindness, n (%)	29 (63.0)	26 (66.67)	3 (42.9)	
VF defect, n (%)	4 (8.7)	3 (7.7)	1 (14.3)	
Visual acuity loss, n (%)	10 (21.7)	8 (20.5)	2 (28.6)	
No symptom	3 (6.5)	2 (5.1)	1 (14.3)	
Mean refractive error (diopter)				
Mean ± SD	−1.31 ± 2.90	−1.41 ± 3.00	−0.64 ± 2.27	0.365
Initial IOP (mmHg)	12.14 ± 3.32	12.34 ± 3.47	11.14 ± 2.44	0.356
Initial BCVA (better seeing eye)				
Mean BCVA(LogMAR)	0.26 ± 0.44	0.29 ± 0.48	0.09 ± 0.08	0.459
Last BCVA (better seeing eye)				
Mean BCVA(LogMAR)	0.46 ± 0.75	0.54 ± 0.80	0.10 ± 0.06	0.302
ERG pattern (Last follow up)^a^				0.004*
Reduced responses, n (%)	12 (35.3)	6 (22.2)	6 (85.7)	
No response, n (%)	22 (64.7)	21 (77.8)	1 (14.3)	
VF pattern (Last follow up)				<0.001*
Normal	2 (4.8)	2 (5.7)	0 (0.0)	
Peripheral constriction, n (%)	4 (9.5)	4 (11.4)	0 (0.0)	
Midperipheral scotoma n (%)	3 (7.1)	2 (5.7)	1 (14.3)	
Central island with pheripheral remnants, n (%)	5 (11.9)	4 (11.4)	1 (14.3)	
Central island, n (%)	23 (54.8)	23 (65.7)	0 (0.0)	
Superior hemisphere, n (%)	5 (11.9)	0 (0.0)	5 (71.4)	

BCVA, best-corrected visual acuity; ERG, electroretinogram; LogMAR, logarithm of the minimum angle of resolution; RHO, rhodopsin; RP, retinitis pigmentosa; SD, standard deviation; VF, visual field.

^a^ Valid n = 34.

*p-value <0.05.

ERG findings at both initial and last visit were documented in 34 patients. All patients with initially preserved central VF showed reduced or no response in photopic and scotopic ERG responses. Generalized RP showed a significantly higher rate of no response in ERG (21 of 27 [77.8%] vs. 1 of 7 [14.3%], *p* = 0.004) and central island patterns in VF (23 of 35 [65.7%] vs. 0 of 7 [0.0%], *p* < 0.001) than that of sector RP.

At the last follow-up visit, the generalized RP group showed a significantly higher rate of visual field impairment progression to late-stage RP than that of the sector RP group (22 of 35 [62.9%] vs. 0 of 7 [0.0%], *p* = 0.003) (Table 2). The generalized RP group showed a higher rate of visual acuity deterioration to late-stage RP than that of the sector RP group, although the *p*-value was not significant (13 of 35 [37.1%] vs. 0 of 7 [0.0%], *p* = 0.079).

**TABLE 2:** Disease progression analysis of BCVA, VF, and CME.

Variables		Total (n = 42)	Generalized RP (n = 35)	Sector RP (n = 7)	*p*-value
VF	(+)	22 (52.4%)	22 (62.9%)	0 (0.0%)	0.003*
	(−)	20 (47.6%)	13 (37.1%)	7 (100.0%)	
BCVA	(+)	13 (31.0%)	13 (37.1%)	0 (0.0%)	0.079
	(−)	29 (61.9%)	22 (62.9%)	7 (100.0%)	
CME	(+)	8 (19.0%)	5 (14.3%)	3 (42.9%)	0.113
	(−)	34 (81.0%)	30 (85.7%)	4 (57.1%)	

VF, visual field; BCVA, best-corrected visual acuity; CME, cystoid macular edema; RP, retinitis pigmentosa.

*p-value <0.05.

VF (+) = central visual field of the better seeing eye less than 10° during follow up.

BCVA (+) = visual acuity of the better seeing eye less than 20/40 during follow up.

CME (+) = cystoid macular edema occurred during follow up.

The generalized RP group showed a lower rate of cystic macular edema (CME) than that of the sector RP group; however, the difference was not statistically significant (5 of 30 [14.3%] vs. 3 of 4 [42.9%], *p* = 0.113) (Table 2; Supplementary Figure S1). Furthermore, no association was observed between CME and any specific pathogenic variants.

**FIGURE 1:**
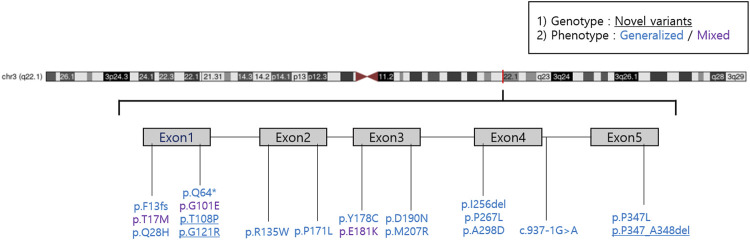
A lollipop diagram of the location of *RHO* mutations in the dataset.

We reported 14 (73.7%) missense mutations, 2 (10.5%) deletion mutations, 1 (5.3%) frameshift mutation, 1 (5.3%) nonsense mutation, and 1 (5.3%) splicing site mutation. Moreover, we identified three novel *RHO* mutations: two missense mutations (p.T108P and p.G121R) and one deletion mutation (p.P347_A348del) (Table 3; Figure 1). All patients harbored a single heterozygous variant of *RHO*. Pathogenic variants were most common in exon 1 (14 of 36 [38.9%]). Of the classified nucleotide changes, 6 of 10 (60%) were identified as either classification 2 or likely classification 2. The most common pathogenic variants were p.P347L (5 of 36 families; 13.8%) and p.T17M (5 of 36 families; 13.8%). p.G101E (4 of 36 families; 11.1%) and p.R135W (4 of 36 families; 11.1%) were the second most common pathogenic variants.

**TABLE 3:** Pathogenic variants included in this cohort of patients of RHO-assisted RP.

Nucleotide change	Amino acid change	Frequency (Families)	Type of mutation(s)	Position	Phenotype	CADD score	Classification	Reported
c.36del	p.F13fs	1(1)	Frameshift	Exon 1	Generalized	25.6	Unclassified	Wang et al. (2019)
c.50C>T	p.T17M	5(5)	Missense	Exon 1	Mixed^a^	24.6	4	Dryja et al. (1991)
c.84G>C	p.Q28H	1(1)	Missense	Exon 1	Generalized	24.6	2	Fernandez-San Jose et al. (2015)
c.190C>T	p.Q64*	1(1)	Nonsense	Exon 1	Generalized	36	Unclassified	Macke et al. (1993)
c.302G>A	p.G101E	4(4)	Missense	Exon 1	Mixed^b^	25.6	Likely 2, 3 or 4	Arai et al. (2015)
c.322A>C	p.T108P	1(1)	Missense	Exon 1	Generalized	20.1	Unclassified	novel
c.361G>C	p.G121R	1(1)	Missense	Exon 1	Generalized	34	Unclassified	novel
c.403C>T	p.R135W	5(4)	Missense	Exon 2	Generalized	26	3	Jacobson et al. (1991)
c.C512 > T	p.P171L	1(1)	Missense	Exon 2	Generalized	24.4	2	Dryja et al. (1991)
c.A533 > G	p.Y178C	5(2)	Missense	Exon 3	Generalized	25.1	2	Farrar et al. (1991)
c.541G>A	p.E181K	2(2)	Missense	Exon 3	Mixed^c^	26.1	2	Dryja et al. (1991)
c.568G>A	p.D190N	2(2)	Missense	Exon 3	Generalized	23.7	2	Keen et al. (1991)
c.620T>G	p.M207R	1(1)	Missense	Exon 3	Generalized	25.7	Unclassified	Farrar et al. (1991)
c.768_770del	p.I256del	1(1)	Inframe deletion	Exon 4	Generalized	20.9	Unclassified	Inglehearn et al. (1991)
c.800C>T	p.P267L	1(1)	Missense	Exon 4	Generalized	29.1	2	Sheffield et al. (1991)
c.893C>A	p.A298D	1(1)	Missense	Exon 4	Generalized	23.1	Unclassified	Kim et al. (2011)
c.937-1G>A		1(1)	Splicing	Exon 4i	Generalized	34	Unclassified	Bell et al. (1994)
c.1040C>T	p.P347L	7(5)	Missense	Exon 5	Generalized	25.7	1	Dryja et al. (1991)
c.1039_1044del	p.P347_A348del	1(1)	Inframe deletion	Exon 5	Generalized	20.5	1	novel

RHO, rhodopsin; RP, retinitis pigmentosa; CADD, combined annotation dependent depletion.

^a^ Four sector RP, and one generalized RP.

^b^ Two sector RP, and two generalized RP.

^c^ One sector RP, and one generalized RP.

**FIGURE 2:**
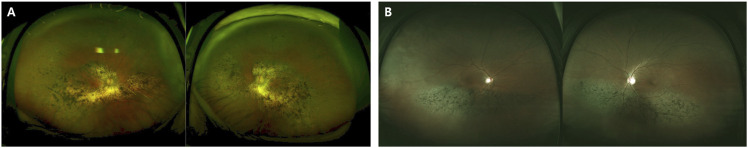
Images of different phenotypes of patients with RP with p.G101E mutations. **(A)** A 78-year-old man carrying the missense mutation (p.G101E). A wide field color fundus photo showing bone-spicule pattern of pigmentary deposits. **(B)** A 61-year-old man carrying the same missense mutation (p.G101E). A wide field color fundus photo showing inferior bone-spicule degeneration fundus pattern of pigmentary deposits.

**FIGURE 3:**
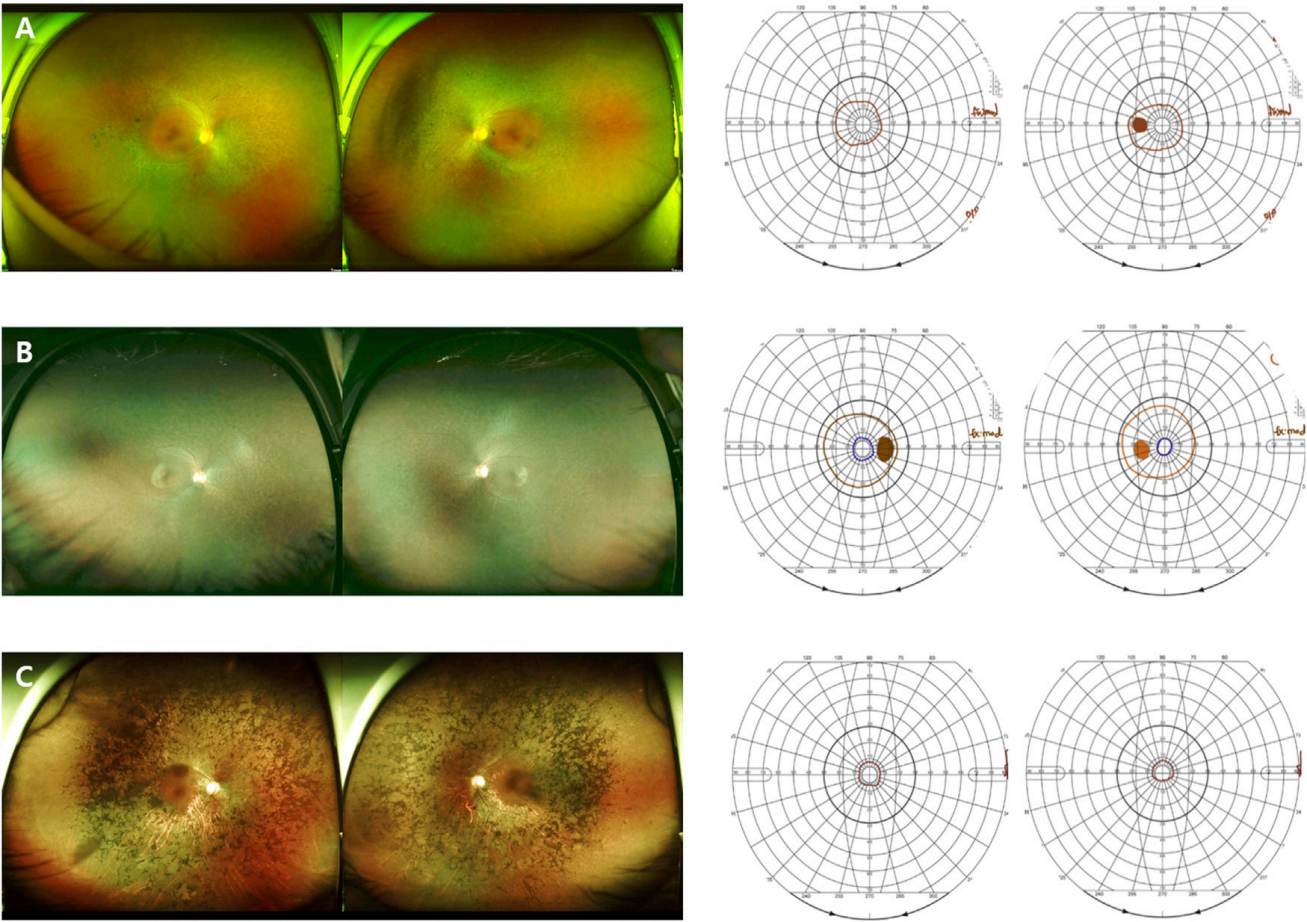
Images of the RP patients with novel RHO mutations. **(A)** A 50-year-old female carrying the missense mutation (p.T108P). A wide field color fundus photo showing bone-spicule pattern of pigmentary deposits in the mid-peripheral retina. The Goldmann visual field test showing a central island on both eyes. **(B)** A 21-year-old female with the missense mutation (p.G121R). A wide field color fundus photo showing some bone-spicule pattern of pigmentary deposits in the mid-peripheral retina. The Goldmann visual field test showing a peripheral constriction field defect. **(C)** A 45-year-old male with the deletion mutation (p.P347_A348del). A wide field color fundus photo showing diffuse bone-spicule pattern of pigmentary deposits. The Goldmann visual field test showing a central island on both eyes.

Patients with the p.T17M variant presented with both the generalized (1 of 5) and sector RP (4 of 5) phenotypes Patients with the p.G101E variant presented with both the generalized (2 of 4) and sector RP (2 of 4) phenotypes (Figure 2). Patients with the p.E181K variant presented with both the generalized RP (1 of 2) and sector RP (1 of 2) phenotypes. During the follow-up period, all patients with sector RP (n = 7) showed bone-spicule pigmentation in the inferior retina, and none of these cases progressed to generalized RP. Three novel mutations (p.T108P, p.G121R, and p.P347_A348del) presented with bone-spicule pattern of pigmentary deposits in the mid-peripheral retina and were classified as generalized RP (Figure 3).

There were no significant differences in the progression to late-stage RP between class A and class B RHO-associated adRP phenotypes for visual field or visual acuity (*p* = 1.000, respectively).

No correlation was observed between the CADD score and the progression of visual field impairment to late-stage RP (Pearson correlation coefficient r = 0.106, *p* = 0.505), as well as the deterioration of visual acuity to late-stage RP (Pearson correlation coefficient r = −0.069, *p* = 0.664).

## 4 Discussion

**FIGURE 4:**
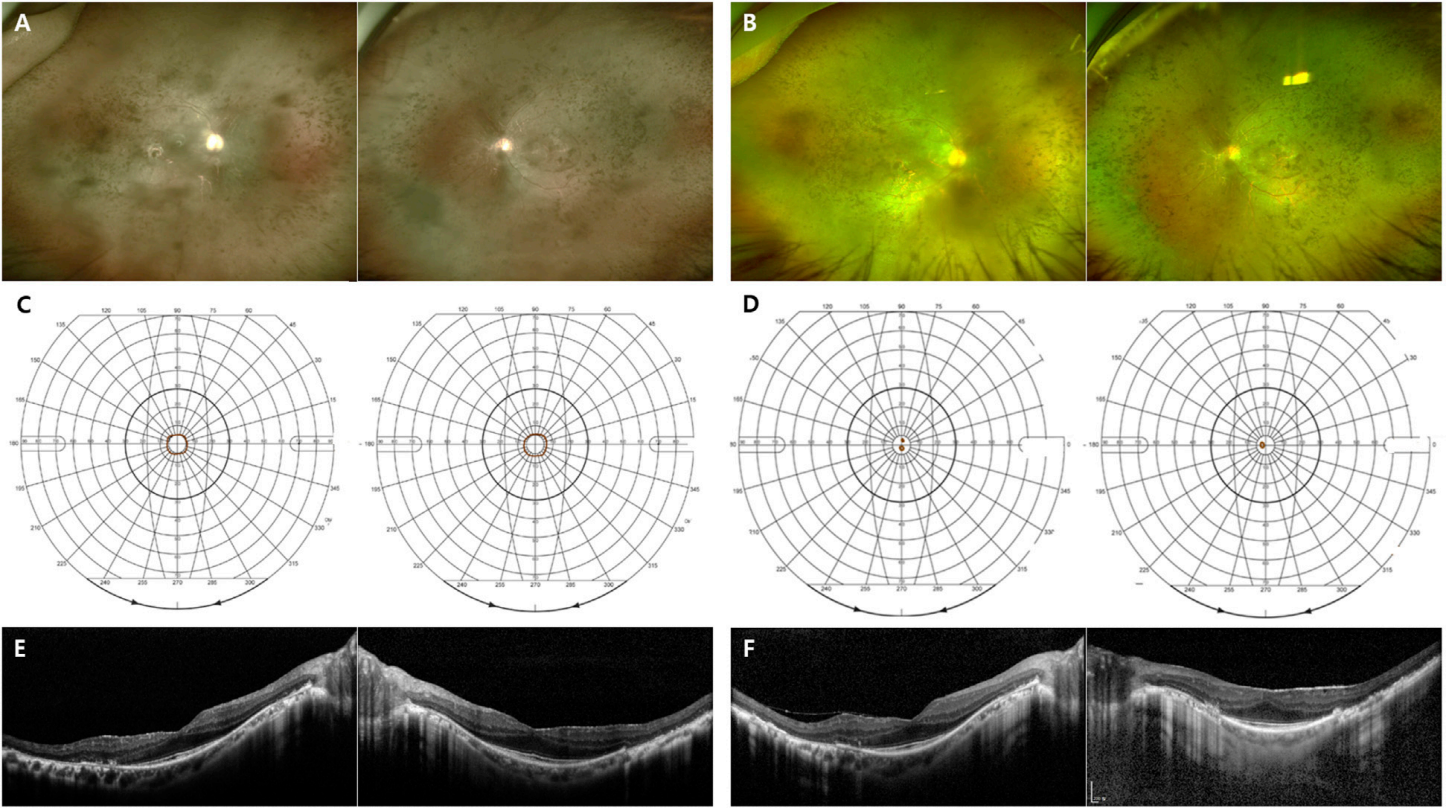
Representative images of the generalized *RHO*-associated RP. **(A–D)** A 48-year-old man carrying the missense mutation (p.R135W). **(A, B)** A wide field color fundus photo at the initial and last visits (11 years gap), respectively. **(C, D)** The Goldmann visual field test at the initial and last visits (11 years gap), respectively. **(E, F)** A 45-year-old man carrying the missense mutation (p.R135W). Horizontal and vertical scans of spectral domain OCT at the initial and last visits (7 years gap), respectively.

**FIGURE 5:**
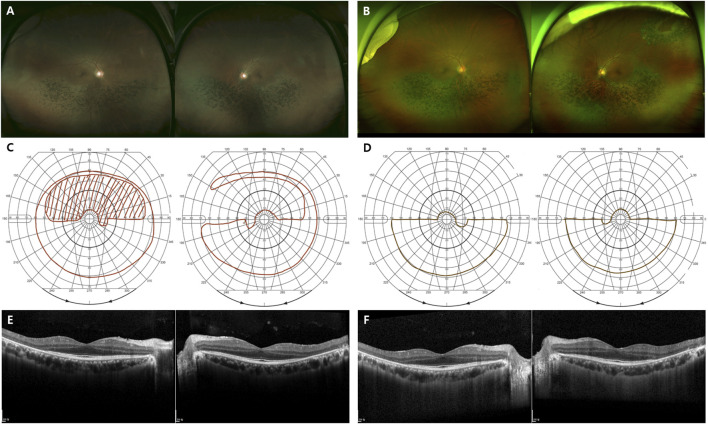
Representative images of the sector *RHO*-associated RP. **(A–F)** A 48-year-old man carrying the missense mutation (p.T17M). **(A, B)** A wide field color fundus photo at the initial and last visits (7 years gap), respectively. **(B)** A barrier laser scar showing at superotemporal area on left eye. **(C, D)** The Goldmann visual field test at the initial and last visits (7 years gap), respectively. **(E, F)** Horizontal and vertical scans of spectral domain OCT at the initial and last visits (7 years gap), respectively.

In this study, we analyzed *RHO*-associated RP cases from multiple centers in Korea and compared the two distinctive clinical patterns: generalized and sector RP. Mutations in the *RHO* gene are associated with autosomal dominant RP (adRP), autosomal dominant congenital stationary night blindness, and autosomal recessive RP (Dryja et al., 1991). Patients with *RHO*-associated adRP experience distinct patterns of retinal dysfunction, either generalized or sector RP, depending on the specific mutation. Generalized RP is characterized by a decrease in rod function across the entire retina, leading to night blindness, peripheral visual field loss, and eventual central vision loss (Figure 4). Sector RP affects one or two quadrants of the retina, primarily the inferior retina (Figure 5). (Berson and Howard, 1971; Berson et al., 1991; Kranich et al., 1993; Van Woerkom and Ferrucci, 2005).

The clinical features and visual prognosis are better in sector RP than in generalized RP. It has been reported that 24 of 26 patients (83.3%) with sector RP retained a BCVA better than 0.3 logarithm of the minimal angle of resolution (LogMAR) (Georgiou et al., 2021). Consistent with this finding, our study showed a mean final logMAR visual acuity of the sector RP group of 0.10, which was better than the 0.54 of the generalized group (Table 2). In addition, a significant difference in the last follow-up visit reported VF pattern was detected between the two groups (*p* < 0.001). No statistically significant difference in visual acuity deterioration during follow-up was observed between the two groups; however, a significant difference in the rate of VF impairment was detected. The significantly higher rate of VF impairment progression to late-stage RP in the generalized RP group was consistent with that of another study (Hartong et al., 2006). However, Nguyen et al. reported a significant difference in visual acuity between the two groups, at the last examination. This discrepancy might have occurred because our study had a relatively small sample size and a short follow-up period. Thus, a few patients had advanced photoreceptor degeneration involving the foveal center.

All patients with sector RP (n = 7) showed bone-spicule pigmentation in the inferior retina, and several studies may explain this result. Generally, the inferior retina is more prone to light exposure, including sunlight and ceiling light, because multiple light sources are oriented upwards. Schwartz et al. (Schwartz et al., 2003) reported that the highest doses of ultraviolet and blue light are located at the superior VF. Tam and Naash et al. (Naash et al., 1996; Tam and Moritz, 2007) reported that the density of apoptotic cells was the highest in the inferior retina and that an increased exposure to a darkened environment delayed retinal degeneration progression in P23H rats. All our study patients with sector RP (seven patients) showed inferior degeneration, which is consistent with the findings of previous studies.

The presence of a central VF does not guarantee normal photopic ERG responses. Even when the central VF is preserved, the photopic ERG responses typically show reduced or no response. Similarly, in our study and other studies of RHO-associated RP, there were no cases of normal photopic ERG responses, regardless of visual field preservation.

Consistent with the findings of the current study (4.8%), the expected frequency of *RHO* mutations in RP has been reported to range from 4% to 7% in previous studies (Chan et al., 2001; Gao et al., 2019). We identified 16 pathogenic variants of *RHO*, including three novel variants. Frequently reported mutations are summarized in Table 4. In the US population, the *RHO* p.P23H mutation is the most common, with a frequency of up to 15% in families with adRP and 61.5% (24 of 39 families) in patients with *RHO*-associated RP (Dryja et al., 1990b; Sung et al., 1991). Moreover, *RHO* p.P347L is a widely reported mutation in the Japanese, Lithuanian, South African, and Spanish populations (Fujiki et al., 1992; Kucinskas et al., 1999; Roberts et al., 2000; Fernandez-San Jose et al., 2015). *RHO* p.R135W is a common mutation in the Han-Chinese family (Wu et al., 2019), and *RHO* p. E181K and p. D190Y are common mutations in the Dutch and Belgian populations (Nguyen et al., 2021). Consistent with previous reports in literature, our study identified p.P347L as one of the two most common pathogenic variants (5 of 36 families; 13.8%). Moreover, we frequently observed the p.T17M variant (5 of 36 families; 13.8%). p.G101E (4 of 36 families; 11.1%) and p.R135W (4 of 36 families; 11.1%) were the second most common variants. These results showed that the variant frequencies largely overlap between different ethnicities, whereas there are several ethnicity-dependent variations such as p.P23H. Pathogenic variants in *RHO* were most frequently detected in exon 1, in 14 of 36 patients (38.9%).

**TABLE 4:** Reports of the frequency of pathogenic variants among RHO-RP families.

Report	No^a^	Ethnic group	Frequency of pathogenic variant
Sung CH, et al. (1991)	39	American	p.P23H (24/39)
			p.R135W (2/39)
Nguyen XT, et al. (2021)	47	Dutch and Belgian	p.D190Y (5/47)
			p.E181K (4/47)
			p.P347L (3/47)
			p.T17M (2/47)
Fernandez-San Jose P et al. (2015)	42	Spanish	p.P347L (9/42)
Fujiki K, et al. (1992)	24	Japanese	p.P347L (4/24)
			p.P23H (1/24)
Yang et al. (2014)	8	Chinese	p.P347L (1/8)
Ziviello et al. (2005)	7	Italian	p.R135W (4/7)
			p.P347L (2/7)
Our study	36	Korean	p.P347L (5/36)
			p.T17M (5/36)
			p.G101E (4/36)
			p.R135W (4/36)

^a^ Number of RHO-RP, families.

We identified three novel *RHO*-associated RP mutations: p.T108P, p.G121R, and p.P347_A348del; their phenotypes were generalized RP, and for p.T108P and p.P347_A348del mutations, the central VF was <10° at the initial and last visits. For the p.G121R mutation case, the central VF was 20–30° at the last visit, possibly because the patient aged 21 years at the last visit, i.e., an early stage of RP.

In our study, the p.T17M, p.G101E, and p.E181K variants showed both generalized and sector RP phenotypes, and none of the variants presented sector RP alone. One possible explanation for this phenomenon is that sector RP eventually progressed to generalized RP in the later stages. Although none of the patients that were initially diagnosed with sector RP progressed to generalized RP, some patients that were initially diagnosed with generalized RP might have started with sector RP. In a previous study, the mutant allele (p.M39R; heterozygous) was transmitted from the affected father to the son, and the phenotype was generalized RP for the father and sector RP for the son (Ramon et al., 2014). Another possibility is that the phenotype could be influenced by other environmental factors such as light exposure. Additional long-term follow-up studies are required to confirm these findings.

*RHO*, comprising 348 amino acids, has a structure divided into three regions: the cytoplasmic, transmembrane, and intradiscal domains (Dryja et al., 1990a; Macke et al., 1993). In this study, seven of seven families (p.T17M, p.G101E, and p.E181K) were diagnosed with sector RP, and these pathogenic variants were located in the intradiscal domain. Consistent with our findings, several studies have reported that *RHO*-associated sector RP is caused by missense mutations; most of which are located in the intradiscal domain (Kranich et al., 1993; Napier et al., 2015; Xiao et al., 2019). Additionally, some sector RP rhodopsin mutations, p.M39R and p.N55K, can also be found in the transmembrane domain (Ramon et al., 2014).

Another approach to the genotype-phenotype correlation of adRP RHO mutations is based on the classification into two major classes in clinical settings (Cideciyan et al., 1998). Class A exhibits early onset severe rod dysfunction, while class B is associated with a later onset and less severe phenotype with slower progression. However, other factors, such as genetic modifiers and the environment, can also influence the disease presentation and may lead to interfamilial variability (Iannaccone et al., 2006). In this study, among eight patients with mutations corresponding to class A (p.P347L or p.P347_A348del), three demonstrated progression to late-stage RP. However, five patients did not show progression to late-stage RP until the last follow-up, suggesting the possible influence of genetic modifiers or environmental factors on disease progression. A patient with p.Q64* corresponding to class B was diagnosed with RP at the age of 13, indicating early onset RP, but only exhibited progression to late-stage RP at the age of 56. Therefore, prospective future studies are necessary to further elucidate the impact of genetic and environmental factors on disease progression.

Many ongoing clinical trials using gene supplementation therapies have been conducted on autosomal recessive and X-linked IRD since the US Food and Drug Administration approved the gene therapy voretigene neparvovec-rzyl (Luxturna). In contrast, autosomal dominant diseases, such as *RHO*-associated RP, are caused by gain-of-function mutations and require a substantially different approach. Targeted gene therapy is promising for *RHO*-associated RP; however, it requires the inhibition of mutant *RHO* protein expression with simultaneously increasing the wild-type-to-mutant *RHO* ratio to effectively reduce the rate of retinal degeneration. Several treatments are currently being developed, including stem cell or retinal tissue transplantation, nutritional supplements, retinal implants, and targeted and non-targeted gene therapies (Meng et al., 2020). Although no universally effective treatment has yet been established for *RHO-*associated RP, understanding and analyzing its genotype, phenotype, and underlying mechanisms can help develop future therapies.

This study had two limitations. First, the sample size was too small to perform a survival analysis. Second, the details of the follow-up protocol were not standardized because of the retrospective design of the study. However, this is the first Korean multicenter study conducted on *RHO*-associated RP that included the largest number of Korean patients with RHO-associated RP.

This multicenter cohort study provided information on the clinical and genetic features of *RHO*-associated RP in Koreans. One of the two most commonly reported pathogenic variants was p.P347L, consistent with the findings of previous reports of non-American populations. Furthermore, we identified p.T17M as a common pathogenic variant. Generalized and sector RP showed different courses of VF and BCVA progression. It is clinically important to expand the genetic spectrum and understand genotype-phenotype correlations to ultimately facilitate the development of gene therapy.

## Data Availability

The datasets presented in this article are not readily available because data obtained from targeted next-generation sequencing or whole exome sequencing are sensitive, and our ethics committee does not authorize the sharing of these data. Requests to access the datasets should be directed to the corresponding authors.

## Abbreviations

BCVA, best-corrected visual acuity; CME, cystic macular edema; D, diopters; ERG, electroretinography; GVF, Goldmann visual field; IOP, intraocular pressure; IRD, inherited retinal dystrophies; LogMAR, logarithm of the minimal angle of resolution; RHO, rhodopsin; RP, retinitis pigmentosa; SD, standard deviation; VF, visual field.
