# Supplementary material for: Clinical and genetic features of Koreans with retinitis pigmentosa associated with mutations in rhodopsin
Source: Front Genet. 2023 Aug 29;14:1240067. doi: 10.3389/fgene.2023.1240067 (PMC10497939; doi:10.3389/fgene.2023.1240067)
Supplement: Supplementary file 1 [file Image1.pdf]

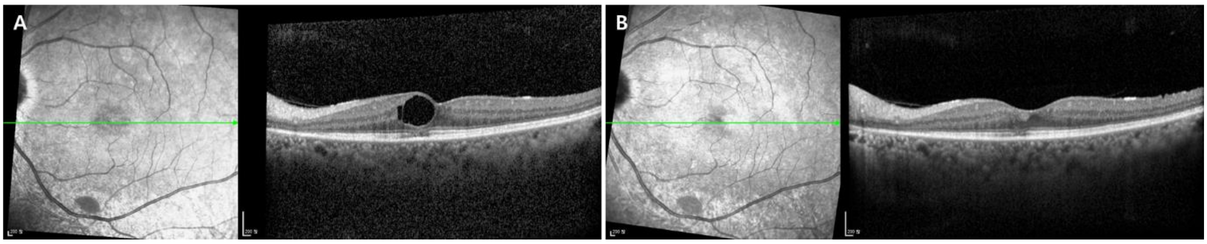

Supplementary figure 1. Representative images of the cystic macular edema (CME). (A) A 78-year-old male carrying the missense mutation (p.G101E). The spectral domain optical coherence test showing CME at initial visit. (B) CME resolved after two intravitreal anti-vascular endothelial growth factor injections and the use of topical steroids and carbonic anhydrase inhibitors.
